# Influenza A (H1N1) 2009 Antibodies in Residents of New South Wales, Australia, after the First Pandemic Wave in the 2009 Southern Hemisphere Winter

**DOI:** 10.1371/journal.pone.0012562

**Published:** 2010-09-07

**Authors:** Gwendolyn L. Gilbert, Michelle A. Cretikos, Linda Hueston, George Doukas, Brian O'Toole, Dominic E. Dwyer

**Affiliations:** 1 Centre for Infectious Diseases and Microbiology, Institute of Clinical Pathology and Medical Research, The University of Sydney, Sydney, New South Wales, Australia; 2 Centre for Epidemiology and Research, New South Wales Department of Health, North Sydney, New South Wales, Australia; 3 School of Public Health, The University of Sydney, North Sydney, New South Wales, Australia; NMRCD, Peru

## Abstract

**Background:**

The first wave of pandemic influenza A(H1N1)2009 (pH1N1) reached New South Wales (NSW), Australia in May 2009, and led to high rates of influenza-related hospital admission of infants and young to middle-aged adults, but no increase in influenza-related or all-cause mortality.

**Methodology/Principal Findings:**

To assess the population rate of pH1N1 infection in NSW residents, pH1N1-specific haemagglutination inhibition (HI) antibody prevalence was measured in specimens collected opportunistically before (2007–2008; 474 specimens) and after (August–September 2009; 1247 specimens) the 2009 winter, and before the introduction of the pH1N1 monovalent vaccine. Age- and geographically-weighted population changes in seroprevalence were calculated. HI antibodies against four recent seasonal influenza A viruses were measured to assess cross-reactions. Pre- and post-pandemic pH1N1 seroprevalences were 12.8%, and 28.4%, respectively, with an estimated overall infection rate of 15.6%. pH1N1 antibody prevalence increased significantly - 20.6% overall - in people born since 1944 (26.9% in those born between 1975 and 1997) but not in those born in or before 1944. People born before 1925 had a significantly higher pH1N1 seroprevalence than any other age-group, and against any seasonal influenza A virus. Sydney residents had a significantly greater change in prevalence of antibodies against pH1N1 than other NSW residents (19.3% vs 9.6%).

**Conclusions/Significance:**

Based on increases in the pH1N1 antibody prevalence before and after the first pandemic wave, 16% of NSW residents were infected by pH1N1 in 2009; the highest infection rates (27%) were among adolescents and young adults. Past exposure to the antigenically similar influenza A/H1N1(1918) is the likely basis for a very high prevalence (49%) of prepandemic cross-reacting pH1N1 antibody and sparing from pH1N1 infection among people over 85 years. Unless pre-season vaccine uptake is high, there are likely to be at least moderate rates including some life-threatening cases of pH1N1 infection among young people during subsequent winters.

## Introduction

The first wave of infection due to pandemic influenza A (H1N1) 2009 - pH1N1 - in Australia began in May, 2009 [1]. There was debate as to whether pH1N1 infections were significantly more prevalent or severe than during an “average” influenza season [2]. Most clinical infections were apparently mild and predominantly affected school children and young adults. Increased numbers of laboratory-confirmed and notified cases, compared with average influenza seasons, could be partly explained by much greater levels of public awareness, medical consultation and laboratory testing [3]. Despite a disease profile generally similar to that of seasonal influenza, pH1N1 infections led to unusually high rates of hospital and intensive care unit (ICU) admissions of relatively young patients with influenza-related illness. ICU admissions for viral pneumonitis were 15 times higher than in previous years and highest in the 25–49 year age-group; 93% of ICU patients were under 65 years [4]. In New South Wales (NSW), the most populous Australian state, syndromic surveillance of emergency department presentations showed unusually high rates of febrile respiratory illness during the 2009 winter but there was neither an increase in deaths attributable to influenza or pneumonia, nor in overall mortality [3].

It is difficult to estimate the true rates of pH1N1 infection or differences between geographic areas and age-groups from limited epidemiological data, but information about population prevalence of infection and immunity is needed to inform vaccine distribution policy and planning for subsequent waves of pH1N1 infection. Serosurveys are used extensively to supplement laboratory notification, hospitalisation and mortality data for many vaccine preventable diseases [5].

The aim of this study was to determine the prevalence of subtype-specific influenza A pH1N1 haemagglutination inhibition (HI) antibodies in a broadly-based sample of children and adult residents of NSW, before and after the first pandemic wave, using opportunistically collected plasma or serum specimens.

## Methods

### Specimens

Clinical chemistry laboratories in NSW were asked to provide serum or lithium heparin-treated plasma specimens which had been submitted for diagnostic testing in August or September, 2009 and would otherwise have been discarded. This period was 3–11 weeks after the first epidemic wave peaked in NSW and before the monovalent pH1N1 vaccine became available. The sample size was calculated to provide power to detect a difference in seroprevalence of 10–15% between age groups with a worst case 95% confidence interval of ±7%. We aimed to test approximately equal numbers of specimens from NSW residents in each of seven age-groups (children: preschool 0–4 years, primary school 5–11 years, secondary school 12–17 years; and adults: 18–34, 35–64 and 65–85 years and 85 years and older), providing a total sample size of ∼1200 specimens. Specimens which represented both urban and rural NSW populations were retrieved using postcode of residence. Specimens were given a unique identifying code and then de-identified; only the sex, age or date of birth and patient postcode were recorded.

To estimate the level of pre-existing antibodies to pH1N1, we also tested stored sera from NSW residents which had been submitted for non-influenza serological testing during 2007 and 2008. The sample size for prepandemic sera was determined largely by availability but we aimed to test approximately 50 in each age-group.

The specimens used for testing had been submitted for diagnostic testing and would otherwise have been discarded. Consistent with longstanding practice in the performance of national serosurveys, informed consent was not obtained from subjects for the use of their specimens in this study. However, all specimens were deidentified before testing and only the age or date of birth and address postcode were recorded. This study, including the waiver of informed consent, was approved by the Sydney West Area Health Service Human Research Ethics Committee.

### Antigens

The antigen used for HI testing was a gamma-irradiated preparation of influenza A/California/07/2009 (pH1N1) virus, provided by the WHO Collaborating Centre for Reference and Research on Influenza, Melbourne, Australia. Antigens derived from four recent seasonal influenza A viruses – Brisbane/59/2007/H1N1, New Caledonia/20/1999/H1N1, Brisbane/10/2007/H3N2, and Wisconsin/67/2005/H3N2 - were used to determine levels of antibody to these viruses in the same specimens.

### Haemagglutination inhibition (HI) assay

A pH1N1 subtype-specific HI assay was developed using established methods. As initial evaluation demonstrated that lithium heparin used as anticoagulant did not significantly affect antibody titres in plasma (compared with serum) whereas EDTA plasma specimens gave less consistent results, only lithium heparin-treated plasma and serum specimens were used.

Briefly, specimens were diluted 1/5 in *Vibrio cholerae* receptor destroying enzyme and incubated overnight at 37°C to remove inhibitors, then diluted 1/2 in citrate and heat inactivated at 56°C. Serial doubling dilutions (and appropriate controls) were reacted with antigens in 96-well V-bottom microtitre trays for 1 hour at room temperature before a 1% v/v suspension of human group O red blood cells was added. After 1–2 hours (or when the cell control had fully haemagglutinated), endpoints were read by two independent operators as the last dilution showing complete inhibition of haemagglutination. Titres of ≥40 were determined to be “positive” for the purpose of this serosurvey.

### Statistical analysis

Geometric mean titres (GMTs) were calculated by assigning a titre of 5 to specimens in which no HI antibody was detected (titre<10). Seroprevalence of pH1N1 antibodies was calculated as the percentage with antibody titres≥40, after weighting for age, sex and geographic region.

The post-pandemic sample size was designed with a disproportionately greater representation of very young and very old persons and with broad coverage of all geographic areas of NSW by using postcode as a stratification variable. As postcodes in NSW are not grouped geographically, there was some disproportionate representation by region. The initial design of the sample required design weights to be calculated to account for the non-uniform probability of selection between age and postcode strata.

In order to compensate for under- and over-sampling from sections of the NSW population, post-stratification weights for both the pre- and post-pandemic samples were also created to balance the sample by five-year age group and by geographic region [6]. Post-stratification weights were calculated using raking since the number of cases in each post-stratification cell was small [7], [8], [9]. Geographic regions were defined using 2006 Australia Standard Geographic Classification of the Australian Bureau of Statistics (ABS) [10]. Statistical Subdivisions within Sydney and Statistical Divisions outside Sydney were used to define the regions. Socioeconomic status was assigned based on the postcode of residence and therefore indicates the socioeconomic status of area of residence not the individual. Socioeconomic status was classified using the Index of Relative Socioeconomic Disadvantage from the Socio-Economic Indexes for Areas (SEIFA) developed by the ABS [11].

Differences in weighted proportions were tested using the Rao-Scott Chi-square test. Confidence intervals for differences in proportions between the pre-pandemic and post-pandemic samples were estimated using bootstrapping with 10,000 replications [12]. Odds ratios were calculated using weighted bivariate logistic regression. SAS version 9.1.3 (SAS Institute Inc., Cary, NC) was used for all weighting and statistical analysis.

## Results

### Pre- and post-pandemic seroprevalence by birth cohort/age-group

A total of 474 “pre-pandemic” specimens collected in 2007–2008 were tested. Antibodies were detected at titres of ≥40 in only one of 53 from children born in 1998 or later. Overall, 12.8% of patients had pre-existing pH1N1 antibody at titres≥40. Differences in pre-pandemic prevalence of pH1N1 antibody titres≥40 across the five youngest birth-cohorts (born after 1944), were not statistically significant but the pre-pandemic prevalence was significantly higher in the 1925–44 birth cohort and higher again in those born before 1925 (Table 1).

**Table 1:** Comparison of prevalence of haemagglutination inhibition assay titres≥40 against influenza A California/07/2009 (pH1N1) collected in pre- and post-pandemic periods, New South Wales, Australia, by year of birth cohort.

Year of birth cohort	Prepandemic (2007–8)				Postpandemic (August–September 2009)				Population change in seroprevalence (95% CI^2^)
	Tested	Titre≥40	GMT	Weighted percent^1^ (95% CI)	Tested	Titre≥40	GMT	Weighted percent^1^(95% CI)	
2005 or after	20	0	5.2	0.0%	207	36	9.6	15.6% (9.9–21.4)	15.6% (10.5–22.4)^3^
1998–2004	33	1	5.3	2.6% (0.0–7.6)	170	27	9.9	12.4% (7.3–17.5)	9.8% (0.0–15.9)^3^
1992–1997	47	6	5.9	5.5% (0.6– 10.4)	176	73	22.9	40.0% (31.0– 49.1)	34.5% (24.0–44.7)^3^
1975–1991	95	12	8.9	15.8% (5.5– 26.0)	229	91	23.0	40.1% (32.7– 47.5)	24.3% (9.6–35.5)^3^
1945–1974	143	9	8.2	6.7% (1.2– 12.2)	231	62	15.4	26.3% (19.8– 32.7)	19.6% (10.1–27.5)^3^
1925–1944	71	26	15.2	33.5% (20.2– 46.8)	171	37	16.9	19.9% (12.9– 26.9)	−13.4% (−30.2–+0.3)
Pre 1925	65	39	33.1	62.3% (49.4–75.2)	63	31	31.8	49.4% (34.5–64.2)	−12.9% (−32.9–+6.7)
Total sample	474	93	8.6	12.8%(8.9–16.6)	1247	357	16.7	28.4% (25.0–31.7)	15.6% (10.2–20.4)

CI = confidence interval.

*Notes:*

1 Weighted by age and geographic region.

2 Bootstrap bias-corrected and accelerated confidence intervals.

3 Statistically significantly (p<0.05).

A total of 1247 “post-pandemic” specimens collected in August and September 2009 were tested. The overall prevalence of pH1N1 antibody titres≥40 in the NSW population, after the first wave of pH1N1 infection, was 28.4% (females 29.0%, males 27.7%; difference not significant). Post-pandemic seroprevalence was highest in those born in the periods 1975–1991 (40.1%), 1992–1997 (40.0%) and pre 1925 (49.4%; Table 1).

**Figure 1:**
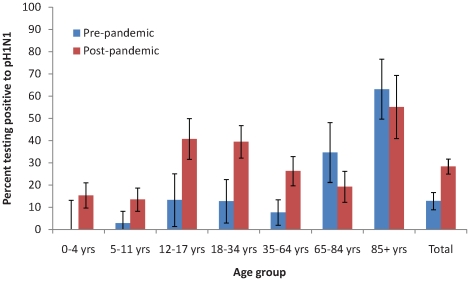
Prevalence of antibody to pandemic influenza A (H1N1) 2009. Data are percentages of subjects with H1N1 haemagglutinating antibody titres≥40, in pre- and post-pandemic samples, from New South Wales residents by age group.

The overall increase in seroprevalence between pre- and post-pandemic periods was 15.6% and differences were statistically significant in birth cohorts after 1944 (those aged less than 65 years; 20.6% change overall), and greatest in the 1992–1997 (aged 12–17; 34.5% change) and 1975–1991 (aged 18–34; 24.3% change) birth cohorts. The increase did not reach statistical significance in the 0–4 and 5–11 age-groups, separately, but when these two groups were combined the increase, from 1.6% (95% CI 0.0–4.7) to 13.7% (95% CI 9.9–17.6); difference 12.1% (p = 0.006, Rao-Scott Chi squared test) was highly significant. Apparent decreases in seroprevalence in the 1925–44 and pre-1925 birth cohorts (65–85 and ≥85 year age-groups) were not statistically significant (Table 1, Figure 1).

### Geographic and socioeconomic differences in pH1N1 seroprevalence

Seroprevalence of pH1N1 antibodies in pre- and post-pandemic specimens were compared between the Sydney region (estimated resident population 4.5 million) and the rest of NSW (population 2.6 million, spread over a very large geographic area; Table 2). There was no significant difference in seroprevalence between regions in pre-pandemic specimens, but the difference in pH1N1 seroprevalence between pre- and post-pandemic specimens was significantly higher in Sydney than in the rest of NSW (19.3% vs 9.6%, Table 2).

**Table 2:** Comparison of prevalence of pH1N1 antibody titres≥40 in specimens collected in pre- and post-pandemic periods from residents of Sydney and the rest of NSW.

Region	Pre-pandemic (2007–8)				Post-pandemic (2009)				Change in seroprevalence (95% CI)^1^	p-value^2^
	Tested	Titre≥40	GMT	Percent (95% CI)	Tested	Titre≥40	GMT	Percent (95% CI)		
**Sydney**	300	61	8.3	11.7 (7.1–16.3)	826	249	17.7	31.0 (26.6–35.5)	19.3% (12.3–25.3)	0.0001
**Rest of NSW**	174	32	9.0	14.5 (7.7–21.4)	421	108	15.2	24.1 (19.1–29.1)	9.6% (0.0–17.2)	0.0438
**Total**	**474**	**93**	**8.6**	**12.8 (8.9–16.6)**	**1247**	**357**	**16.7**	**28.4 (25.0–31.7)**	**15.6% (10.2–20.4)**	**0.0001**

*Notes*.

1 Bootstrap bias-corrected and accelerated confidence intervals.

2 Rao-Scott Chi-square test. Numbers are weighted by age and region.

Results were also analysed by accessibility/remoteness index of Australia (ARIA+) categories. Seroprevalence was highest (30.2%) in the most accessible (urban) region, but differences by accessibility classification were not significant (p = 0.30). Finally, seroprevalence was compared across socioeconomic indices for areas (SEIFA) quintiles, based on postcode of residence. Seroprevalence varied from 25.5% to 33.3% by socioeconomic status, but differences were not statistically significant (p = 0.58).

### Levels of antibodies to pH1N1 and other recent seasonal influenza A viruses

In addition to pH1N1 antibody, subtype/strain-specific HI antibodies against four recent seasonal influenza A virus antigens were measured in postpandemic specimens. The highest seroprevalences (at titres≥40) for different viruses were in different age-groups/birth cohorts: 5–11 (1998–2004) and 12–17 year olds (1992–1997) for Brisbane/59/2007/H1; 5–11 (1998–2004), 12–17 (1992–1997) and 18–34 year olds (1975–1991) for New Caledonia/20/1999/H1; 12–17 (1992–1997) for Wisconsin/67/2005/H3 and 5–11 (1998–2004) for Brisbane10/2007/H3. There were significant differences in seroprevalence and GMTs across age groups for each of the five viruses (Table 3).

**Table 3:** Proportion with subtype-specific HI antibodies and geometric mean titres against five different influenza A subtypes in specimens collected in August/September 2009, New South Wales, Australia, by age-group.

		Influenza A virus subtype									
Year of birth cohort	No. tested	California/072009/H1 (pH1N1)		Brisbane/592007/H1		New Caledonia/20/ 1999/H1		Wisconsin/2005/H3		Brisbane/102007/H3	
		Titre≥40n (%)	GMT	Titre≥40n (%)	GMT	Titre≥40n (%)	GMT	Titre≥40n (%)	GMT	Titre≥40 n (%)	GMT
2005 onwards	207	36 (16)	9.6	17 (7)	7.9	9 (4)	6.5	10 (5)	7.1	10 (4)	6.8
1998–2004	170	27 (12)	9.9	67 (38)	21.3	46 (26)	13.4	27 (17)	9.4	38 (19)	9.7
1992–1997	176	73 (40)	22.9	52 (27)	15.3	29 (16)	10.6	41 (19)	10.8	4 (5)	6.3
1975–1991	229	91 (40)	23.0	38 (18)	12.7	34 (16)	11.2	29 (13)	10.0	6 (4)	7.4
1945–1974	231	62 (26)	15.4	19 (8)	9.1	15 (6)	8.3	16 (6)	8.1	9 (4)	7.8
1925–1944	171	37 (20)	16.9	22 (13)	11.2	18 (9)	9.9	28 (16)	14.0	26 (16)	11.8
Pre 1925	63	31 (49)	31.8	8 (10)	11.3	3 (4)	8.1	12 (16)	15.5	8 (11)	10.3
**Total**	1247	357 (28)	16.7	223 (15)	11.4	154 (11)	9.6	163 (11)	9.5	101 (7)	8.1

GMT = geometric mean titre.

Differences in prevalence of positive titres (using Chi-squared test for trend) and in GMTs (using ANOVA on “raw” titres) between age-groups are statistically significant for all five virus antigens (p<0.001).

The proportions of specimens with HI antibody titres≥40 against seasonal influenza A subtypes were compared for specimens with pH1N1 antibody titres≥40 vs <40 (Table 4). There was no significant relationship between the presence or absence of pH1N1 antibody and that of any seasonal influenza A virus antibody, except that samples in which pH1N1 antibody was detected were less likely to have Brisbane/59/2007/H3 antibody than pH1N1 antibody negative samples; this difference was just statistically significant (odds ratio 1.8; p = 0.05).

**Table 4:** Proportions of specimens with HI antibodies (with titres≥40) against four seasonal influenza A viruses in pH1N1 antibody positive (titre≥40) and negative samples.

California/07/2009/H1 (pH1N1) result:	Brisbane/59/2007/H1	New Caledonia /20/1999/H1	Wisconsin/2005/H3	Brisbane/10/2007/H3
Negative<40	16.3%	11.1%	11.7%	8.0%
Positive≥40	12.4%	11.7%	9.7%	4.5%
OR (95% CI); P value	1.38 (0.88–2.19); 0.16	0.95 (0.59–1.52); 0.82	1.23 (0.74–1.21); 0.42	**1.80 (1.00–3.32); 0.05**

CI = confidence interval; OR = odds ratio.

## Discussion

During the southern hemisphere winter of 2009, in Australia, the pH1N1 epidemic period lasted about 18 weeks in all [1], [3]. However, there were considerable variations in rates of spread, numbers of laboratory-confirmed cases, timing of epidemic peaks and rates of hospital and ICU admission between and within different States and major cities [1], [3], [4]. Most cases apparently occurred in school-aged children and were mild and generally not laboratory-confirmed. Hospital admission rates were highest in the 0–4 year and 50–59 year age-groups, but lower than for an average influenza season among adults over 60 years [3]. Around one third of patients admitted to ICU with pH1N1 influenza-related illness had no underlying risk factors, most required mechanical ventilation and at least 14% died [4].

In view of these unusual features, estimates of true infection rates in different age-groups and geographic areas are needed to inform immunisation policy and planning for the predicted second wave of infection. Clinical and epidemiological data alone are inadequate, since mild or asymptomatic infections were not recorded and the clinical presentation and rates vary in different age groups. Laboratory diagnostic strategies also varied across States and at different times within the pandemic period. Although pH1N1 was the most common influenza virus detected, seasonal influenza A/H3N2 and A/H1N1 (and other respiratory viruses) were circulating at the same time, at least early in the pandemic [13]. A seroprevalence survey, encompassing all age-groups and geographic areas, is a timely and practicable source of more comprehensive data.

Influenza A serosurveillance is complicated by cross-reactions between different influenza A subtypes, variable and often relatively short-lived influenza antibody responses, repeated previous infections and the technical challenges of HI and viral neutralization assays [14]. We have shown previously [5] that, despite some theoretical disadvantages, the opportunistic sampling strategy used in this study can produce results comparable with those from randomly collected samples (which also have disadvantages) for serosurveillance of many vaccine-preventable diseases. To improve representativeness of this sampling strategy, we employed an age- and geographically-based sampling frame for the post-pandemic sera, and adopted post-stratification sampling weights to achieve better representation of the NSW population structure. This serosurvey is the first reported from the southern hemisphere, where the pH1N1 epidemic coincided with the usual winter influenza and respiratory virus season. The results confirmed many of the epidemiological features of this outbreak as shown by notification and hospitalisation data.

Genomic and proteomic studies show that pH1N1 is most like the North American swine A/H1N1 and pandemic A/H1N1(1918) viruses and distinct from recent seasonal A/H1N1 and other 20^th^ century pandemic influenza A viruses [15], [16], [17]. Host-specific genomic signatures of pH1N1, which are mainly swine-like, show a high level of identity with influenza A/H1N1(1918). Since the beginning of the 2009 pandemic, some mutations have occurred in functional viral genes, which may reflect adaptation to humans and/or changes in virulence, but there is no evidence of antigenic change [17].

Similarities between pH1N1 and influenza A/H1N1(1918) have been reflected in a number of surveys of sera collected before the 2009 pandemic [16], [18], [19]. Few, if any, children and fewer than 10% of young adults had cross-reacting pH1N1 neutralising antibody at titres≥40 whereas at least one third of adults aged >65 years had significant titres. While seasonal influenza vaccination produced many-fold increases in GMT of seasonal A/H1N1 antibodies it resulted in a modest, two-fold increase in GMT of cross-reactive pH1N1 antibody in young adults and none in children or adults ≥60 years [18], [19]. These results are consistent with other evidence that pH1N1 and seasonal A/H1N1 are antigenically different, but suggest that, nevertheless, seasonal H1N1 vaccination may boost low levels of pre-existing, cross-reactive pH1N1 antibody.

This is supported by the results of a serosurvey in an unvaccinated population in southern China, in which no cross-reacting pH1N1 antibody was detected in prepandemic sera in subjects aged ≥60 years, in a largely unimmunised population; pH1N1 antibody levels did not increase after administration of seasonal influenza vaccine. The authors suggested that the presence of cross-reactive pH1N1 antibody in western populations could be due to repeated seasonal influenza vaccination, rather than exposure to older, seasonal H1N1 influenza viruses [19], as suggested previously [20].

The significantly higher proportions of subjects over 85 years with cross-reacting antibodies in pre-pandemic specimens in our study, are consistent with results of recent studies from England and Finland [21], [22]. In England, the proportion of samples with cross-reacting HI antibodies with titres≥1∶32 ranged from 1·8% in children aged 0–4 years to 31·3% in adults aged 80 years or older. The trends were similar in Finland, where 96% of people born before 1919 (at least 90 years old) had cross-reacting pH1N1 antibodies, compared with 14–77% of those born between 1920 and 1944 and very few of those born since 1944. It is likely that A/H1N1(1918)-like viruses were the first influenza A viruses to which many individuals born during or before the 1920s were exposed and that repeated exposure, subsequently, to seasonal influenza A viruses and/or vaccines, has boosted immunity to A/H1N1(1918) and hence cross-reactive immunity to pH1N1. This would account for the sparing of these age-groups from significant levels of influenza infection during the current pandemic.

We demonstrated an overall increase in the seroprevalence of pH1N1 antibodies with titres≥40 (corresponding with infection rates) of nearly 16% in NSW residents following the southern hemisphere 2009 winter. Increases were restricted to age-groups less than 65 years, particularly the 12–17 (1992–7 birth cohort) and 18–34 (1975–91) year age groups with increases of 34.5% and 24.3%, respectively. In a similar study, Miller *et al* showed comparable age-related differences in seroprevalence increases in London and the West Midlands, where infection rates were highest, but increases only in children under 15 years in other areas [21]. In Singapore, seroconversion (or infection) rates in four adult cohorts were: 29% in military personnel; 13.5% in the general population; 6.5% in hospital staff and only 1% in long-term care residents. Older age was associated with reduced seroconversion rates [23]. In our study, the proportional increase in seroprevalence (or infection) in the Sydney area (19.3%), was twice that detected in the rest of NSW (9.6%). In combination with data from England and Singapore, this indicates – not surprisingly - that higher infection rates occur in denser populations.

In addition to pH1N1-specific antibodies, we also studied the prevalence, in post-pandemic specimens, of antibodies to four recent seasonal viruses, including two A/H1N1 and two A/H3N2 strains. In general the highest seroprevalence rates and GMTs were in the same young age-groups, in which pH1N1 antibodies were most prevalent, although there was some variation between strains. However, a major difference was the relatively high seroprevalence of pH1N1 antibody compared with the other four recent seasonal influenza A viruses in those aged over 85 years (and to a lesser extent in the 65–85 year group). This supports the hypothesis that repeated infection by seasonal influenza A viruses or vaccination boost responses to the first influenza A virus to which these older people were exposed when very young - which is likely to have been A/H1N1(1918)-like viruses - rather than to more recent seasonal viruses. The proposition that recent seasonal influenza A virus vaccine can provide modest protection against pH1N1, despite antigenic differences, has been suggested previously [20], [24], [25], but disputed by others [26], [27]. Our observation that there was a small but significant negative association between the presence of pH1N1 antibodies and seasonal Brisbane/59/2007A/H3N2 antibodies, in post-pandemic samples, may be a chance finding but, alternatively, could support this proposition.

In summary, we have shown that the highest rates of infection were among children over 12 years and young to middle-aged adults. The elderly were largely spared, confirming clinical and epidemiological data. As expected, infection rates were higher in Sydney than in smaller cities and rural areas of NSW. The study provided evidence of previous exposure to an antigenically similar influenza A virus in the oldest age-groups (>85 years and 65–85 years). The overall infection rates in NSW indicate that pH1N1 infections are likely to recur with at least moderate frequency in younger age-groups during the 2010 winter influenza season, unless there is high pH1N1 immunisation uptake.
